# Clinical Presentation, Risk Factors, and Treatment Modalities of Hepatocellular Carcinoma: A Single Tertiary Care Center Experience

**DOI:** 10.1155/2016/1989045

**Published:** 2016-07-25

**Authors:** Abdulrahman A. Aljumah, Hadi Kuriry, Mohammed AlZunaitan, Mohammed Al Ghobain, Mohamed Al Muaikeel, Ashwaq Al Olayan, Fahad Azzumeea, Bader Almutairi, Abduljaleel AlAlwan, Hamdan AlGhamdi

**Affiliations:** ^1^Division of Hepatology, Department of Hepatobiliary Sciences, King Abdulaziz Medical City, Ministry of National Guard Health Affairs, P.O. Box 22490, Riyadh 11426, Saudi Arabia; ^2^King Saud bin Abdulaziz University for Health Sciences, P.O. Box 22490, Riyadh 11426, Saudi Arabia; ^3^Department of Medicine, King Abdulaziz Medical City, Ministry of National Guard Health Affairs, P.O. Box 22490, Riyadh 11426, Saudi Arabia; ^4^Department of Medical Imaging, King Abdulaziz Medical City, Ministry of National Guard Health Affairs, P.O. Box 22490, Riyadh 11426, Saudi Arabia; ^5^Department of Adult Medical Oncology, King Abdulaziz Medical City, Ministry of National Guard Health Affairs, P.O. Box 22490, Riyadh 11426, Saudi Arabia

## Abstract

*Objective*. To investigate the risk factors, clinical characteristics, treatment modalities, and outcomes in Saudi patients with HCC and propose points for early detection of the disease.* Methods*. Patients were stratified according to underlying risk factors for the development of HCC. Barcelona Clinic Liver Cancer (BCLC) was used for cancer staging. Treatment was classified into surgical resection/liver transplantation; locoregional ablation therapy; transarterial embolization; systemic chemotherapy; and best supportive care.* Results*. A total of 235 patients were included. Males had higher tumor size and incidence of portal vein thrombosis. Viral hepatitis was a risk factor in 75.7%. The most common BCLC stages were B (34.5%) and A (33.6%), and the most common radiological presentation was a single nodule of less than 5 cm. Metastases were present in 13.2%. Overall, 77 patients (32.8%) underwent a potentially curative treatment as the initial therapy. The most commonly utilized treatment modality was chemoembolization with 113 sessions in 71 patients. The overall median survival was 15.97 ± 27.18 months.* Conclusion*. HCC in Saudi Arabia is associated with high prevalence of HCV. Potentially curative therapies were underutilized in our patients. Cancer stage BCLC-B was the most frequent (34.5%) followed by BCLC-A (33.6%). The overall median survival was shorter than other studies.

## 1. **Introduction**

Hepatocellular carcinoma (HCC) is a primary liver malignancy and one of the most common cancers worldwide and is one of the leading causes of cancer-related death [1–3]. Worldwide, it represents around 90% of the total primary liver cancer [4].

It is considered as the fifth most common cancer in men (554,000 cases, 7.5% of the total) and the ninth in women (228,000 cases, 3.4%). The prognosis for liver cancer is very poor which has made it globally the second most common cause of death from cancer and was estimated to be accountable for approximately 746,000 deaths in 2012 (9.1% of the total) [5].

The highest liver cancer rates are found in East and Southeast Asia and in Middle and Western Africa [6].

This difference in incidence of liver cancer between different geographical regions and countries is mainly attributed to difference in the incidence of underlying risk factors: viral hepatitis, alcohol use, occupational exposure; and nonalcoholic fatty liver disease (NAFLD) [7–12].

HCC is secondary to liver cirrhosis in 80% of patients and is the main cause of death in liver cirrhosis patients in Europe [13]. Only 30–40% of patients present with early-stage disease amenable to curative treatments, such as resection or liver transplantation (LT), while others can only undergo local therapies or palliative care [13].

In Saudi Arabia, according to the most recent cancer registry, HCC accounts for 4.8% of all newly diagnosed cases of all types of cancers. It ranked fourth in males and eighth in females with a male to female ratio of 2.1 : 1 and it accounts for 83% and 80% of all liver cancers in males and females, respectively [14]. However it is probable that these numbers were greatly underestimated the accurate incidence, because previously, the national registry depends on a tissue biopsy for confirming the diagnosis of HCC, which was not performed to substantial number of patients with highly suspected diagnosis of HCC and hence they were not counted. This is no longer required for the diagnosis of HCC except for certain cases as per current national and international practice guidelines [1, 4, 15].

The objective of this study is to investigate the possible risk factors, clinical characteristics, treatment modalities, and outcomes in Saudi patients with HCC and propose points for consideration that may help in the early detection of the disease.

## 2. **Materials and Methods**

This is a retrospective cohort study of all cases with presumed or confirmed diagnosis of HCC who presented to King Abdulaziz Medical City (KAMC) in Riyadh, Saudi Arabia, between January 2009 and September 2011. The criteria of HCC diagnosis were based on current published local guideline [15] that included (1) diagnosis by liver tumor biopsy and (2) cirrhotic liver, with lesion larger than 1 cm in diameter, and at least one imaging modality (dynamic CT-scan or MR) has confirmed early arterial enhancement and venous washout of the lesion.

Data were collected from the patient's medical records which include patient demographics, comorbidities, date of HCC diagnosis, date of death, and date of last follow-up, presentation of HCC, etiology of underlying liver disease, presence of cirrhosis, tumor number (HCC were classified according to their numbers into solitary lesion, two lesions, and multiple lesions (3 or more lesions)), size (according to the maximal tumor diameter and in cases of multinodular tumors, the largest one was measured), presence of portal vein tumor thrombosis (PVTT), presence of vascular invasion and extrahepatic spread, features of portal hypertension including esophageal varices, ascites and splenomegaly, biochemical and hematological parameters, modality of first intervention, and patient survival (measured in months).

The diagnosis of cirrhosis was based on histopathology or combination of imaging features (including hepatic nodularity and ascites), poor synthetic liver function, and findings suggestive of cirrhosis on clinical examination.

Patients were stratified according to underlying risk factors for liver cirrhosis and subsequent development of HCC. Risk factors include HBV, HCV, HBV, and HCV coinfection, alcohol, nonalcoholic fatty liver disease, autoimmune hepatitis (AIH), schistosomiasis, or cryptogenic liver cirrhosis. Cancer staging was based on the recently updated Barcelona Clinic Liver Cancer (BCLC) staging system and treatment strategy [4].

All patients received an initial therapy that was appropriate for their tumor stage and general conditions. The choice of therapy was according to the decision of our institutional tumor board which is a multidisciplinary team that comprises all of the corresponding subspecialties and in view of the local and international guidelines. To properly assess the types of initial therapy, treatment methods were divided into six forms: (1) surgical resection, (2) locoregional ablation therapy, percutaneous ethanol injection (PEI), and radiofrequency ablation (RFA), (3) embolization, transarterial chemoembolization (TACE), and transarterial radioembolization (TARE), (4) systemic chemotherapy and Sorafenib, (5) liver transplantation, and (6) best supportive care. If the patient did not receive any therapy, the initial treatment method was considered as best supportive care.

Posttherapy follow-up as per protocol was performed in all patients and was intended to detect suboptimal response to initial therapy.

In case of suboptimal response to initial therapy, residual or early recurrence, or new primary HCC, patient will be offered the most suitable therapy option sequentially, as second or third intervention to control the tumor and improve patient outcome.

Survival and mortality were investigated by examination of the medical record for the last clinic visit or hospital admission. Survival was defined as the interval between the date of HCC diagnosis and either the date of liver-related death or the last follow-up to 12 September 2012.

### 2.1. Statistical Analysis

Descriptive statistics were displayed as percentage or continuous data with mean and standard deviation. Fisher's exact test or the Chi square test were used to compare frequencies to assess group differences for categorical variables and Student's *t*-test was used to compare means to assess differences between continuous variables. Comparison of nonparametric data was performed by Mann-Whitney *U* test. Survival curves were estimated using the Kaplan-Meier method and the differences in the survival rates between the groups were compared by the log-rank test. A *P* value of <0.05 was considered statistically significant. Statistical Package for Social Sciences (SPSS) version 19.0 (SPSS, Chicago, IL, USA) was used for data analysis.

## 3. Results

Medical records of 253 patients were reviewed. Eighteen patients were excluded because they had metastatic nonprimary liver tumor and did not meet HCC diagnostic criteria or incomplete medical records. A total of 235 patients were included. Majority were male (*n* = 168, 71.5%) with proportion of male to female patients of 2.5 : 1. The mean age upon initial diagnosis was (65 ± 10). Detailed demographic and clinical characteristics are shown in Tables 1 and 2. The male subjects had a significantly higher mean age (66.1 ± 10.8 versus 62.9 ± 9.8, *P* = 0.028), tumor size (6.5 ± 5.6 versus 3.7 ± 2.7, *P* = 0.000), platelets (211.5 ± 142.2 versus 174 ± 112.8), and incidence of portal vein thrombosis (18.5% versus 7.5%, *P* = 0.035) but a lower mean body mass index (26.7 ± 5.4 versus 29.7 ± 6.5, *P* = 0.001). Male gender was associated with multifocal tumor (three or more) (*P* = 0.019) and HCC on noncirrhotic liver (*P* = 0.049) compared to female.

Statistically there was no significant difference between males and females in most of other variables, including comorbidities, initial presentation, underlying etiology of liver disease, or liver status as shown in Table 3. The most common stage at presentation was BCLC-B (34.5%) followed by BCLC-A (33.6%) (Table 2).

At the time of diagnosis, most HCC patients were symptomatic (52.3%). Symptomatic cases tend to present commonly with abdominal pain, distension, and anorexia.

Concerning the underlying etiology of liver disease as HCC risk factors, viral hepatitis was the commonest (75.7%) followed by cryptogenic liver disease (21.7%).

In our cohort the most common pattern of radiological presentation of HCC was a single nodule of less than 5 cm in diameter in 74 cases (31.5%). Extrahepatic metastases were present in 31 patients (13.2%) with lung parenchyma metastasis being the most common site 19/31 (61.3%).

Treatment modalities utilized in our patients at different BCLC stages are shown in Tables 4 and 5. Overall, 77 (32.8%) of our investigated patients underwent a potentially curative treatment (RFA, PEI, resection, or LT) as the initial therapy. Noncurative therapy (TACE, TARE, and Sorafenib) and best supportive care were offered to 71 patients (30.2%) and 87 (37%) patients as initial therapy, respectively. Among treatment modalities, the most commonly utilized method for tumor control was chemoembolization with 113 sessions in 67 patients. Only 3 cases underwent LT as the first intervention modality. However, most of transplanted patient (18 Patients) received an initial local ablation therapy or chemoembolization and then underwent LT during follow-up as a subsequent intervention. During follow-up, our patients received 2-3 sessions (maximum of 5 sessions) of intervention on average before their tumor becomes under control.

From the gender point of view, 40 (59.7%) female patients underwent local ablative therapy or embolization as an initial therapy, compared to 76 (45%) male patients. No significant rate difference in relation to gender was observed for patients who have received best supportive care (34.3% and 38.1% for female and male, resp.) or LT (1.5% and 1.20% for female and male, resp.) as the initial therapeutic modality. Twenty male patients received Sorafenib as initial treatment; however, none of the female patients received so (Table 3). Ultimately, Sorafenib was utilized in 25 males and 3 females during all stages of intervention.

**Figure 1:**
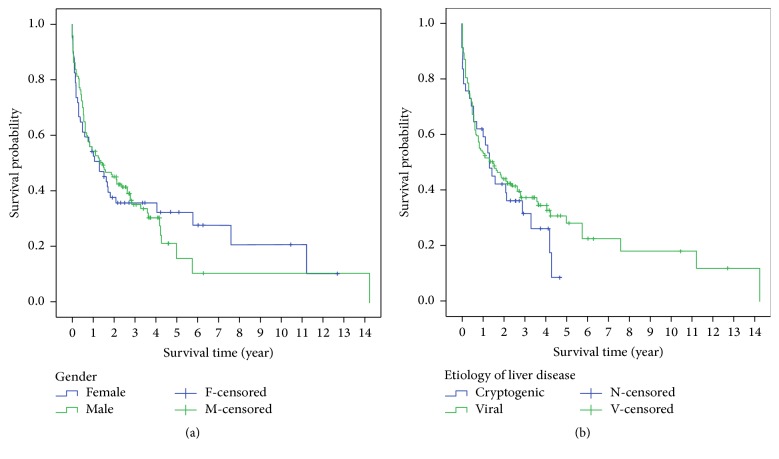
(a) Cumulative survival rate in patients with hepatocellular carcinoma by gender. (b) Cumulative survival rate in patients with hepatocellular carcinoma by etiology of liver disease.

At the time the survival analysis was performed for censored cases, a total of 125 patients (53.2%) were identified as dead. The cumulative survival rate at 1 and 3 years in male and female groups was 65.5% and 54.2% versus 64.2 and 47.8%, respectively. There was no significant difference in overall survival outcome between the two groups (*P* = 0.926) (Figure 1(a)). Similarly, there was no significant difference between cryptogenic and viral hepatitis liver disease as the underlying etiology on the overall survival outcome of HCC (*P* = 0.374) (Figure 1(b)). However, there was a significant difference in survival outcome based on treatment modalities. The overall survival rate at 1 and 3 years in transplanted patients was 100%. The overall survival rate at 1 year in patients who had surgical resection was 100%, while in patients who have received RFA/PEI, TACE/TARE, and Sorafenib it was 82.8%, 78.8%, and 70%, respectively. The three-year survival rate in patients who had surgical resection or Sorafenib was around 55% while, in patients who have received RFA/PEI and TACE/TARE, the survival rate was 73.4% and 67.3%, respectively. Patients with best supportive care had the lowest survival rate at 1 and 3 years 37.9% and 25.3%, respectively. According to BCLC tumor stage, 1-year survival was 100%, 81.8%, 67.9%, 41.5%, and 32% in stages 0, A, B, C, and D, respectively. While 3-year survival rate was 87.5% in stage 0, it was 70.1%, 51.2%, 31.7%, and 24% in stages A, B, C, and D, respectively. The overall median survival from diagnosis was 15.97 ± 27.18 months (range from 0 to 172.3 months). Mean survival time for males was 26.37 months ± 34.83 and for females was 22.03 months ± 22.83. However, this difference did not reach statistical significance (*P* = 0.941). We performed univariate and multivariate analysis of factors associated with poor survival outcome (Table 6). On multivariate analysis, age, BCLC stages C and D, multifocal tumor, AST, and ALT correlated with poor outcome.

## 4. Discussion

HCC is an important worldwide health issue, particularly in regions where viral hepatitis prevalence is high. Several reports have identified clearly the magnitude of viral hepatitis in Saudi Arabia [16–19]. However, there are only limited reports on the health burden of HCC and the available modalities of its treatment.

This study revealed that HCC was more prevalent in males, which is in agreement with figures from the previous Saudi Cancer Registry report [14], as well as other local and regional studies [11, 20–24]. The causes for gender differences in the incidence may be explained in part by the differences in exposure to carcinogens like smoking and alcohol and also to the commonly higher rate of viral hepatitis in men [25]. On the other hand, genetic and hormonal factors have been postulated as risk factors. Estrogen inhibits IL-6 production by Kupffer cells and subsequently decrease cells injuries and proliferation in women while testosterone promotes liver cells in men [26–28].

The peak age of incidence of HCC was found to be in older population with mean age 65 years, which is in line with the recently published data from Saudi Arabia [22] and countries like Italy [29], Japan [30], and Portugal [31]. However, this is fairly different from the average age as reported in the latest US literature, which was between 57 and 59 years [32–34]. In contrast to studies of Alswat et al. [22] and Buch et al. [35], females tend to be younger in age with cirrhotic liver and smaller tumor size than male.

Our data showed that chronic viral hepatitis was the major risk factor contributing to the development of HCC and majority were related to HCV infection, which is similar to recent report [22]. Hepatitis B was the second risk factors for HCC in our study; however, this is quite different from what was reported from developed countries [36–39]. Remarkably, alcohol as a risk factor for underlying liver disease has contributed to only minority of patients (<1%).

The clinical presentation of HCC in our patients was not different from those in other studies [23, 40], with abdominal pain and abdominal distension being the commonest symptoms. This presentation generally reflects the problem of late presentation.

In our study, the morphological pattern of HCC at the time of diagnosis was unifocal in almost half of the cases, in keeping with other retrospective studies [24, 29, 41], but the contrary was true in the recent report from regional study [11, 22, 23, 40].

With regard to liver function, the majority of our population has preserved liver function, Child-Turcotte-Pugh (CTP) score, class A, which is consistent with other studies [24, 29, 41, 42]. In terms of HCC staging, BCLC combined stages A and B comprise nearly 70% of cases, similar to Fenoglio et al. [29], but more than reported by Marrero et al. [34].

The development of HCC on underlying liver cirrhosis was noted in 81.3% of the patients. Singal et al. in a recent meta-analysis found that the rate of presence of underlying cirrhosis between studies was ranging from 32.4% to 100%, with a pooled rate of 90.9% [43].

The incidence of extrahepatic metastases has been reported in up to 42% [44]. Distant metastases were found in 13.2% of our patients and occurred mainly to the lungs, similar to other studies [22, 45]. In contrast other retrospective reports have revealed much lower rate of metastases [29, 34, 46]. In our cohort, we attributed this relatively high rate of distant metastases to late presentation, delayed referral from other centers, and the aggressive cancer biology. Portal vein thrombosis is a critical issue that can deteriorate the prognosis of HCC because it can lead to wide dissemination of tumors through the liver and cause a marked deterioration of hepatic function. Portal vein thrombosis was documented in 15.3% of the cases in our data consistent with low incidence reported from regional and European studies [22, 29, 40], compared to high incidence reports [34, 45–47]. We observed that male patients tend to have higher rate of portal vein thrombosis, similar to Buch et al. [35].

Notably, potentially curative therapies were underutilized and 37% of our HCC patients received only supportive care for their symptoms, which is higher than some reports [34, 45]. We attributed this to the unsuitable general condition of the patients for advanced therapy, presence of poor prognostic factors, and aggressive behavior of HCC. However our best supportive care rate is considered much lower than most of studies that have reported considerably higher rates of palliative therapy [32, 33, 48–50].

With regard to gender, in our study, a larger percentage of female patients (60%) underwent local ablative or embolization therapy, compared to male patients and almost similar percentage received best supportive care or LT, which is inconsistent with Devaki et al. result [32]. Exclusively males received Sorafenib as initial treatment while Shah et al. [48] and Zak et al. [49] report no gender difference in intervention.

LT had a limited role in the therapy of HCC in our population. Despite a relatively large number of potentially LT-eligible patients (90 patients; 8 patients in BCLC-0 and 79 patients in BCLC-A, as shown in (Table 2) in addition to three more patients who were downstaged from BCLC-B and BCLC-C to BCLC-0/A after subsequent interventions), out of those, only 21 patients (23.3%) were transplanted, giving a total transplant rate in our cohort of 8.9%, which is less than what is reported by others [32]. This may be due to the fact that the donor pool for LT in Saudi Arabia is limited.

In our population, the overall median survival was shorter than other studies [29, 31–34]. The condition of the patients with regard to the underlying liver disease, tumor burden, and comorbidities could have influenced the prognosis. Other factors like socioeconomic status, access to care, and cultural barriers that were not evaluated in this study could have played a role in the overall outcomes.

There is conflicting published data regarding the impact of gender and etiology on HCC patient survival and prognosis. Our data confirmed that neither gender nor etiology of liver disease has contributed to the survival rate of HCC patients (Figure 1(a)). Similar findings have been obtained in previous studies [22, 29, 31, 32, 49, 50].

We have made extensive efforts to include larger number of HCC patients with more detailed data. However, our study being hospital-based and from a single tertiary care center has some limitations including possible selection bias as we receive cases from various areas of the country. In addition, we used a retrospective design; therefore, it was possible that many patients were lost to follow-up and we could not collect enough data about their outcome. One further limitation is unavailability of data about treatment modality, indication, complication, and outcome. Despite these limitations, our study highlighted the need for further prospective studies to identify the prognostic factors in HCC patients and to appropriately screen these patients in anticipations of an earlier diagnosis and, therefore, better outcome.

In summary, we believe that our findings are closely representing the actual unique picture of HCC in Saudi Arabia with high prevalence of HCV and older patients with advanced HCC cases. Significant differences in the receipt of therapy were also observed by gender and underlying liver disease. These data suggest the important role of early screening and aggressive management of chronic viral hepatitis particularly hepatitis C, which is potentially possible in the era of the new direct acting antiviral treatment. Further studies are needed to help in describing factors that contribute to low rate of curative therapy, like access to care, surveillance, treatment refusal, lack of specialized care centers, and physician awareness of newly available therapies.

## Figures and Tables

**Table 1:** General demographic data of hepatocellular carcinoma (HCC) patients.

	Mean ± SD	*n* (%)
Age (years)	65 ± 10	
BMI	27.6 ± 5.9	
Gender		
(male : female)		(2.5 : 1)
Male		168 (71.50%)
Hypertension		123 (52.30%)
Diabetes		136 (57.90%)
Dyslipidemia		30 (12.80%)
Common symptoms		
Abdominal pain		66 (28.10%)
Abdominal distension		35 (14.90%)
Anorexia		30 (12.80%)
Fatigue		27 (11.50%)
Weight loss		20 (8.50%)
Jaundice		18 (7.70%)
Underlying cause of liver disease		
Viral hepatitis		178 (75.70%)
(i) *HCV*		*110 (46.80%)*
(ii) *HBV *		*62 (26.40%)*
(iii) *HBV + HCV*		*6 (2.60%)*
Nonviral hepatitis		57 (24.30%)
(i) *Cryptogenic*		*51 (21.70%)*
(ii) *Autoimmune*		*3 (1.30%)*
(iii) *Schistosomiasis*		*2 (0.90%)*
(iv) *Alcohol*		*1 (0.40%)*

SD: standard deviation; BMI: body mass index; HBV: hepatitis B virus; HCV: hepatitis C virus.

**Table 2:** Clinical characteristics of hepatocellular carcinoma (HCC) patients.

	Mean ± SD	(*n*, %)	
Tumor size (cm)	5.7 + 5.1		
*Number of tumors*			
Single		117	(49.80%)
Two lesions		33	(14.00%)
Multiple (≥3)		85	(36.20%)
*Child-Turcotte-Pugh score*			
A		135	(57.40%)
B		75	(31.90%)
C		25	(10.60%)
Portal vein thrombosis		36	(15.30%)
Vascular invasion		28	(11.90%)
Metastasis		31	(13.20%)
Noncirrhotic liver		44	(18.70%)
Liver transplant		21	(8.9%)
MELD	11 + 5		
Bilirubin, *μ*mol/L	41.5 ± 113		
Albumin, g/L	34 ± 7		
AST, U/L	96 ± 103		
ALT, U/L	64 ± 59		
INR	1.2 ± 0.4		
AFP	10934.2 ± 48		
Platelets, ×10^9^/L	201 ± 135		
*BCLC stages*			
*Stage 0* (very early)		8	(3.4%)
*Stage A* (early)		79	(33.6%)
*Stage B* (intermediate)		81	(34.5%)
*Stage C* (advanced)		42	(17.9%)
*Stage D* (terminal)		25	(10.6%)

SD: standard deviation; MELD: model for end-stage liver disease; AFP: alpha-fetoprotein; AST: aspartate aminotransferase; ALT: alanine aminotransferase; INR: international normalized ratio; BCLC: Barcelona Clinic Liver Cancer.

**Table 3:** Comparison of demographic characteristics, tumor factors, and treatment modalities between male and female patients.

	Female *n* (%) mean ± SD		Male *n* (%) mean ± SD		*P* value
Hypertension	41	61.20%	82	48.80%	0.086
Diabetes	42	62.70%	94	56.00%	0.345
Underlying cause of liver disease					
Viral hepatitis	56	83.60%	122	72.60%	0.077
Nonviral hepatitis	11	16.40%	46	27.40%	
Symptoms at presentation	36	53.70%	87	51.80%	0.787
Noncirrhotic liver	7	10.40%	37	22.00%	0.049
Number of tumors					
Single	33	49.30%	84	50.00%	0.019
Two lesions	15	22.40%	18	10.70%	
Multiple (≥3)	19	28.40%	66	39.30%	
Metastasis	5	7.50%	26	15.50%	0.101
Vascular invasion	7	10.40%	21	12.50%	0.661
Child-Turcotte-Pugh score					
A	31	46.30%	104	61.90%	0.08
B	26	38.80%	49	29.20%	
C	10	14.90%	15	8.90%	
BCLC stages					
Stage 0	2	3.00%	6	3.60%	0.011
Stage A	32	47.80%	47	28.00%	
Stage B	15	22.40%	66	39.30%	
Stage C	8	11.90%	34	20.20%	
Stage D	10	14.90%	15	8.90%	
Portal vein thrombosis	5	7.50%	31	18.50%	0.035
Initial intervention					
Best supportive care	23	34.30%	64	38.10%	0.013
Chemoembolization	13	19.40%	39	23.20%	
Ablation therapy	27	40.30%	37	22.00%	
Sorafenib	0	0.00%	20	11.90%	
Surgical resection	3	4.50%	6	3.60%	
Transplant	1	1.50%	2	1.20%	
AGE	63 ± 10		66 ± 11		0.028
BMI	29.75 ± 6.51		26.75 ± 5.38		0.001
Tumor size (cm)	3.69 ± 2.67		6.53 ± 5.63		0.000
Bilirubin, *μ*mol/L	31.1 ± 29.8		45.7 ± 132.5		0.181
Albumin, g/L	31 ± 7		34 ± 7		0.002
AST, U/L	92 ± 77		97 ± 112		0.723
ALT, U/L	58 ± 42		67 ± 65		0.204
INR	1.4 ± 0.7		1.2 ± 0.2		0.018
Creatinine	90 ± 91.7		90 ± 49.3		0.997
AFP	13128.1 ± 75		10059.3 ± 31		0.752
Platelets, ×10^9^/L	174 ± 113		212 ± 142		0.035
MELD	11.9 ± 5.9		11.1 ± 4.7		0.312

SD: standard deviation; BMI: body mass index; MELD: model for end-stage liver disease; AFP: alpha-fetoprotein; AST: aspartate aminotransferase; ALT: alanine aminotransferase; INR: international normalized ratio; BCLC: Barcelona Clinic Liver Cancer.

**Table 4:** Intervention modalities at different stages.

	1st intervention (*n* = 235)	2nd intervention (*n* = 88)	3rd intervention (*n* = 43)	4th intervention (*n* = 26)	5th intervention (*n* = 13)
TACE	17.40%	35.20%	44.20%	53.80%	53.80%
TARE	4.30%	5.70%	7%	7.70%	0.00%
RFA	16.20%	13.6%	11.60%	3.80%	0.00%
PEI	11.50%	13.6%	18.6%	19.20%	7.70%
Resection	3.80%	2.30%	0.00%	0.00%	0.00%
Transplant	1.30%	13.6%	11.60%	0.00%	7.70%
Sorafenib	8.50%	2.30%	7%	0.00%	23.10%
Best supportive care	37%	13.60%	0.00%	15.40%	7.70%

TACE: transarterial chemoembolization; TARE: transarterial radioembolization; RFA: radiofrequency ablation; PEI: percutaneous ethanol injection.

**Table 5:** Initial intervention modalities per BCLC tumor stage.

Initial intervention	BCLC				
	0	A	B	C	D
PEI	5	20	1	0	1
RFA	2	26	5	0	5
TACE	0	22	19	0	0
TARE	0	2	7	1	0
Sorafenib	0	0	11	9	0
Best supportive care	1	5	31	32	18
Resection	0	3	6	0	0
Transplant	0	1	1	0	1

BCLC: Barcelona Clinic Liver Cancer; TACE: transarterial chemoembolization; TARE: transarterial radioembolization; RFA: radiofrequency ablation; PEI: percutaneous ethanol injection.

**Table 6:** Univariate and multivariate cox regression analysis of variables associated with poor survival in HCC patient.

	Univariate analysis				Multivariate analysis			
	*P* value	HR	95.0% CI		*P* value	HR	95.0% CI	
			Lower	Upper			Lower	Upper
Symptomatic cases	0	2.157	1.501	3.101				
Nonviral hepatitis	0.022	1.617	1.073	2.437				
Multiple tumor (≥3)	0.00	2.996	2.019	4.446	0.000	2.679	1.577	4.552
Metastasis	0.00	5.195	3.242	8.324				
Vascular invasion	0.00	3.849	2.25	6.586				
CTP (B)	0.00	2.67	1.813	3.93				
CTP (C)	0.00	4.31	2.531	7.339				
BCLC (C)	0.00	18.776	4.382	80.445	0.044	6.283	1.052	37.545
BCLC (D)	0.001	10.921	2.51	47.511	0.000	2.679	1.577	4.552
Portal vein thrombosis	0.00	4.923	3.064	7.91				
AGE	0.00	1.039	1.019	1.06	0.000	1.048	1.023	1.074
AST	0.00	1.004	1.003	1.005	0.000	1.006	1.003	1.009
ALT	0.031	1.002	1	1.005	0.001	0.993	0.989	0.997
BMI	0.024	0.964	0.934	0.995				
Tumor size	0.00	1.078	1.045	1.111				
Albumin	0.00	0.947	0.922	0.972				
Creatinine	0.033	1.002	1	1.004				
Platelets	0.006	1.002	1.001	1.003				
MELD	0.00	1.069	1.045	1.094				

CTP: Child-Turcotte-Pugh score; BCLC: Barcelona Clinic Liver Cancer; MELD: model for end-stage liver disease; AST: aspartate aminotransferase; ALT: alanine aminotransferase; BMI: body mass index.
